# Dicyclo­hex­yl(4-isopropyl­phen­yl)phosphane selenide

**DOI:** 10.1107/S1600536812004643

**Published:** 2012-02-10

**Authors:** Sizwe Makhoba, Alfred Muller, Zanele Phasha

**Affiliations:** a Research Center for Synthesis and Catalysis, Department of Chemistry, University of Johannesburg, PO Box 524, Auckland Park, Johannesburg 2006, South Africa

## Abstract

In the title compund, C_21_H_33_PSe, the Se=P bond is part of a distorted tetra­hedral environment on the P atom. Both cyclo­hexyl groups adopt chair conformations. A cone angle of 170° was calculated using an adaptation of the Tolman model. Inter­molecular C—H⋯Se and C—H⋯*Cg* contacts are observed (*Cg* is the centroid of the benzene ring).

## Related literature

For background studies aimed at understanding the transition metal–phospho­rus bond, see: Muller *et al.* (2008 ▶); Roodt *et al.* (2003 ▶). For transition metal complexes with PCy_2_(4-^*i*^Pr—C_6_H_4_), see: Makhoba *et al.* (2011 ▶); Vuba & Muller (2012 ▶). For background to cone angles, see: Tolman (1977 ▶).
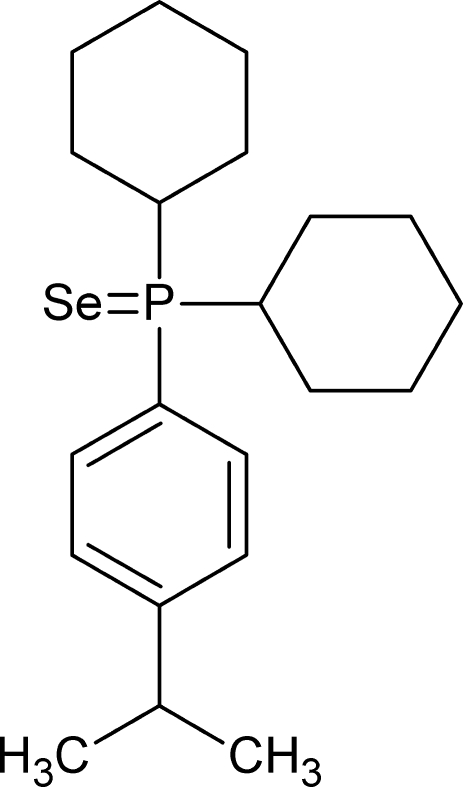


## Experimental

### 

#### Crystal data

C_21_H_33_PSe
*M* *_r_* = 395.4
Monoclinic, 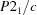

*a* = 13.1311 (10) Å
*b* = 13.6991 (10) Å
*c* = 11.7821 (8) Å
β = 103.106 (2)°
*V* = 2064.2 (3) Å^3^
*Z* = 4
Mo *K*α radiation
μ = 1.90 mm^−1^
*T* = 100 K
0.22 × 0.14 × 0.1 mm

#### Data collection

Bruker APEX DUO 4K CCD diffractometer
Absorption correction: multi-scan (*SADABS*; Bruker, 2008 ▶) *T*_min_ = 0.681, *T*_max_ = 0.833
28000 measured reflections
5140 independent reflections
4397 reflections with *I* > 2σ(*I*)
*R*_int_ = 0.040

#### Refinement

*R*[*F* ^2^ > 2σ(*F* ^2^)] = 0.023
*wR*(*F* ^2^) = 0.057
*S* = 1.02
5140 reflections
210 parameters
H-atom parameters constrained
Δρ_max_ = 0.41 e Å^−3^
Δρ_min_ = −0.24 e Å^−3^

### 

Data collection: *APEX2* (Bruker, 2011 ▶); cell refinement: *SAINT* (Bruker, 2008 ▶); data reduction: *SAINT* and *XPREP* (Bruker, 2008 ▶); program(s) used to solve structure: *SIR97* (Altomare *et al.*, 1999 ▶); program(s) used to refine structure: *SHELXL97* (Sheldrick, 2008 ▶); molecular graphics: *DIAMOND* (Brandenburg & Putz, 2005 ▶); software used to prepare material for publication: *WinGX* (Farrugia, 1999 ▶).

## Supplementary Material

Crystal structure: contains datablock(s) global, I. DOI: 10.1107/S1600536812004643/kp2386sup1.cif

Structure factors: contains datablock(s) I. DOI: 10.1107/S1600536812004643/kp2386Isup2.hkl

Supplementary material file. DOI: 10.1107/S1600536812004643/kp2386Isup3.cml

Additional supplementary materials:  crystallographic information; 3D view; checkCIF report

## Figures and Tables

**Table 1:** Hydrogen-bond geometry (Å, °) *Cg*1 is the centroid of the C13–C18 benzene ring.

*D*—H⋯*A*	*D*—H	H⋯*A*	*D*⋯*A*	*D*—H⋯*A*
C1—H1⋯Se1^i^	1.00	3.09	4.0500 (14)	162
C9—H9*B*⋯*Cg*1^ii^	0.99	2.81	3.6471	143

Symmetry codes: (i) 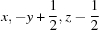; (ii) 
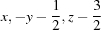
.

**Fig. 1.**
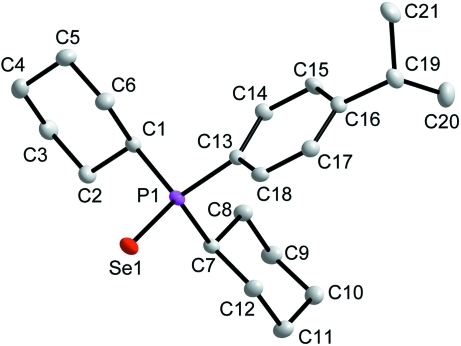
A view of the title compound showing the numbering scheme of atoms and displacement ellipsoids (drawn at the 50% probability level). H atoms omitted for clarity.

**Fig. 2.**
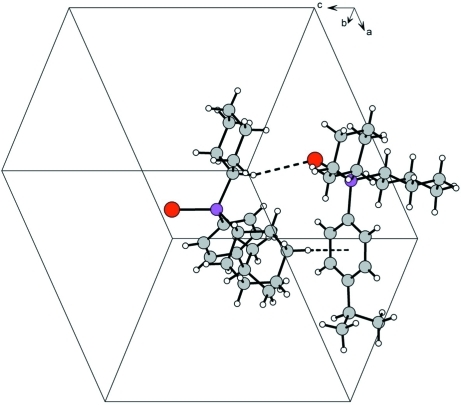
Packing diagram showing the interactions observed for the structure.

## supplementary crystallographic information

### Comment

The bonding of phosphorus to transitional metals have being investigated extensively, with several attempts to divide the properties of the phosphorus ligand into steric and electronic effects. Various techniques such as single-crystal X-ray crystallography, multi nuclear NMR and IR (Roodt *et al.*, 2003) have been used to this extent. Recently we have also included selenium derivatives of the phosphorus compounds into this study (Muller *et al.*, 2008). This route seems viable as the use of expensive transition metals and steric influence from other ligands in the coordination sphere are eliminated, leaving only crystal packing effects as an additional influence on the steric property of the phosphorus ligand. As part of this investigation we report here the selenium derivative of PCy_2_(4-^i^Pr—C_6_H_4_) where Cy = cyclohexyl and ^i^Pr = isopropyl.

Molecules of the title compound (Fig. 1) adopts a distorted tetrahedral arrangement about the P atom with average C—P—C and Se—P—C angles of 106.0° and 112.7° respectively. The cone angle was found to be 170° when the Se—P distance was adjusted to 2.28 Å (the default value from Tolman, 1977). This value is *ca* 5° larger than previous reported values where the present phosphine was bonded to a transition metal centre (Makhoba *et al.*, 2011; Vuba & Muller, 2012). This indicates to some extend the flexibility of this phosphine ligand and its ability to use space to enable less crowding of its substituents. Weak intermolecular C—H···Se and C—H···*Cg* contacts are observed (Table 1, Fig. 2) and link the molecules as infinite chains in the [001] direction.

### Experimental

KSeCN (10 mg, 0.0694 mmol) and PCy_2_(4-^i^Pr—C_6_H_4_) (21.96 mg, 0.0694 mmol) were both dissolved in a minimum amount of methanol (10–20 ml). The KSeCN solution was added drop wise (5 min) to the phosphine solution while stirring at room temperature. The final solution was left to evaporate slowly in order to give crystals that are suitable for single-crystal X-ray study.

### Refinement

All H atoms were positioned in geometrically idealised positions with C—H = 1.00 Å, 0.99 Å, 0.98 Å and 0.95 Å for methine, methylene, methyl and aromatic H atoms respectively and constrained to ride on their parents atoms with *U*_iso_(H) = 1.2*U*_eq_, except for methyl where *U*_iso_(H) = 1.5*U*_eq_ was utilized. The initial positions of methyl H atoms were located from a Fourier difference map and refined as fixed rotor. The highest residual electron density of 0.41 e Å^-3^ was located 0.76 Å from C19, and the deepest hole of -0.24 e Å^-3^ is 0.81 Å from P1. Both represent no physical meaning.

### Crystal data

**Table:**

C_21_H_33_PSe	*F*(000) = 832
*M**_r_* = 395.4	*D*_x_ = 1.272 Mg m^−^^3^
Monoclinic, *P*2_1_/*c*	Mo *K*α radiation, λ = 0.71073 Å
Hall symbol: -P 2ybc	Cell parameters from 8561 reflections
*a* = 13.1311 (10) Å	θ = 2.3–28.2°
*b* = 13.6991 (10) Å	µ = 1.90 mm^−^^1^
*c* = 11.7821 (8) Å	*T* = 100 K
β = 103.106 (2)°	Cuboid, colourless
*V* = 2064.2 (3) Å^3^	0.22 × 0.14 × 0.1 mm
*Z* = 4	

### Data collection

**Table:**

Bruker APEX DUO 4K CCD diffractometer	5140 independent reflections
Graphite monochromator	4397 reflections with *I* > 2σ(*I*)
Detector resolution: 8.4 pixels mm^-1^	*R*_int_ = 0.040
φ and ω scans	θ_max_ = 28.3°, θ_min_ = 1.6°
Absorption correction: multi-scan (*SADABS*; Bruker, 2008)	*h* = −17→17
*T*_min_ = 0.681, *T*_max_ = 0.833	*k* = −18→18
28000 measured reflections	*l* = −15→15

### Refinement

**Table:**

Refinement on *F*^2^	Primary atom site location: structure-invariant direct methods
Least-squares matrix: full	Secondary atom site location: difference Fourier map
*R*[*F*^2^ > 2σ(*F*^2^)] = 0.023	Hydrogen site location: inferred from neighbouring sites
*wR*(*F*^2^) = 0.057	H-atom parameters constrained
*S* = 1.02	*w* = 1/[σ^2^(*F*_o_^2^) + (0.0262*P*)^2^ + 0.6415*P*] where *P* = (*F*_o_^2^ + 2*F*_c_^2^)/3
5140 reflections	(Δ/σ)_max_ = 0.001
210 parameters	Δρ_max_ = 0.41 e Å^−^^3^
0 restraints	Δρ_min_ = −0.24 e Å^−^^3^

### Special details

**Table:**

Experimental. The intensity data was collected on a Bruker Apex DUO 4K CCD diffractometer using an exposure time of 10 s/frame. A total of 5967 frames were collected with a frame width of 0.5° covering up to θ = 28.31° with 100% completeness accomplished.Analytical data: ^31^P {H} NMR (CDCl_3_, 160 MHz): δ = 54.1 (s, 1P)
Geometry. All e.s.d.'s (except the e.s.d. in the dihedral angle between two l.s. planes) are estimated using the full covariance matrix. The cell e.s.d.'s are taken into account individually in the estimation of e.s.d.'s in distances, angles and torsion angles; correlations between e.s.d.'s in cell parameters are only used when they are defined by crystal symmetry. An approximate (isotropic) treatment of cell e.s.d.'s is used for estimating e.s.d.'s involving l.s. planes.
Refinement. Refinement of *F*^2^ against ALL reflections. The weighted *R*-factor *wR* and goodness of fit *S* are based on *F*^2^, conventional *R*-factors *R* are based on *F*, with *F* set to zero for negative *F*^2^. The threshold expression of *F*^2^ > σ(*F*^2^) is used only for calculating *R*-factors(gt) *etc*. and is not relevant to the choice of reflections for refinement. *R*-factors based on *F*^2^ are statistically about twice as large as those based on *F*, and *R*-factors based on ALL data will be even larger.

### Fractional atomic coordinates and isotropic or equivalent isotropic displacement parameters (Å^2^)

**Table:**

	*x*	*y*	*z*	*U*_iso_*/*U*_eq_	
P1	0.63459 (3)	0.16154 (3)	0.07287 (3)	0.01280 (8)	
Se1	0.589654 (11)	0.097347 (10)	0.218666 (12)	0.01702 (5)	
C1	0.53424 (10)	0.24106 (10)	−0.01384 (12)	0.0147 (3)	
H1	0.564	0.2718	−0.0762	0.018*	
C2	0.43736 (11)	0.18232 (11)	−0.07302 (13)	0.0193 (3)	
H2A	0.4569	0.1344	−0.1272	0.023*	
H2B	0.4107	0.1458	−0.0132	0.023*	
C3	0.35114 (11)	0.24925 (11)	−0.14041 (14)	0.0228 (3)	
H3A	0.375	0.2799	−0.206	0.027*	
H3B	0.2881	0.2099	−0.1733	0.027*	
C4	0.32313 (12)	0.32877 (12)	−0.06224 (15)	0.0239 (3)	
H4A	0.2693	0.3722	−0.1089	0.029*	
H4B	0.2936	0.2985	−0.0005	0.029*	
C5	0.41921 (12)	0.38870 (11)	−0.00652 (14)	0.0222 (3)	
H5A	0.3998	0.4382	0.046	0.027*	
H5B	0.4452	0.4234	−0.068	0.027*	
C6	0.50595 (11)	0.32336 (11)	0.06286 (13)	0.0193 (3)	
H6A	0.4826	0.2944	0.1296	0.023*	
H6B	0.5688	0.3634	0.0942	0.023*	
C7	0.66780 (10)	0.06797 (10)	−0.02535 (12)	0.0139 (3)	
H7	0.6056	0.0244	−0.0499	0.017*	
C8	0.69309 (11)	0.11074 (10)	−0.13626 (13)	0.0174 (3)	
H8A	0.6317	0.1473	−0.1804	0.021*	
H8B	0.7523	0.157	−0.1145	0.021*	
C9	0.72156 (12)	0.02992 (11)	−0.21352 (13)	0.0198 (3)	
H9A	0.6606	−0.0136	−0.24	0.024*	
H9B	0.7393	0.0594	−0.2833	0.024*	
C10	0.81410 (12)	−0.02969 (11)	−0.14730 (13)	0.0205 (3)	
H10A	0.8764	0.0128	−0.1253	0.025*	
H10B	0.8301	−0.0824	−0.1981	0.025*	
C11	0.78950 (12)	−0.07406 (11)	−0.03796 (13)	0.0206 (3)	
H11A	0.8515	−0.11	0.0058	0.025*	
H11B	0.7313	−0.1213	−0.0606	0.025*	
C12	0.75906 (11)	0.00468 (10)	0.04060 (12)	0.0173 (3)	
H12A	0.8203	0.0469	0.0712	0.021*	
H12B	0.7388	−0.0269	0.1078	0.021*	
C13	0.75153 (10)	0.23630 (10)	0.11383 (12)	0.0137 (3)	
C14	0.77795 (11)	0.30400 (10)	0.03631 (12)	0.0159 (3)	
H14	0.7302	0.3173	−0.0356	0.019*	
C15	0.87363 (11)	0.35183 (10)	0.06399 (13)	0.0171 (3)	
H15	0.8906	0.3976	0.0106	0.021*	
C16	0.94567 (10)	0.33377 (10)	0.16937 (12)	0.0160 (3)	
C17	0.91711 (11)	0.26870 (10)	0.24719 (13)	0.0167 (3)	
H17	0.9639	0.2567	0.3201	0.02*	
C18	0.82115 (11)	0.22069 (10)	0.22028 (12)	0.0150 (3)	
H18	0.8031	0.1769	0.2751	0.018*	
C19	1.05187 (11)	0.38265 (11)	0.19607 (13)	0.0193 (3)	
H19	1.0879	0.3646	0.2775	0.023*	
C20	1.11914 (12)	0.34511 (13)	0.11461 (15)	0.0278 (4)	
H20A	1.1266	0.2741	0.1225	0.042*	
H20B	1.1884	0.3757	0.1354	0.042*	
H20C	1.0855	0.3616	0.0339	0.042*	
C21	1.04331 (12)	0.49378 (11)	0.19028 (14)	0.0241 (3)	
H21A	1.0042	0.5134	0.1126	0.036*	
H21B	1.1135	0.5223	0.2055	0.036*	
H21C	1.0068	0.5169	0.249	0.036*	

### Atomic displacement parameters (Å^2^)

**Table:**

	*U*^11^	*U*^22^	*U*^33^	*U*^12^	*U*^13^	*U*^23^
P1	0.01478 (16)	0.01252 (16)	0.01143 (17)	−0.00245 (12)	0.00365 (13)	−0.00016 (13)
Se1	0.02188 (8)	0.01700 (8)	0.01373 (8)	−0.00395 (6)	0.00727 (5)	0.00076 (5)
C1	0.0143 (6)	0.0151 (7)	0.0144 (7)	−0.0018 (5)	0.0029 (5)	0.0004 (5)
C2	0.0165 (6)	0.0179 (7)	0.0223 (8)	−0.0026 (5)	0.0017 (6)	−0.0022 (6)
C3	0.0170 (7)	0.0237 (8)	0.0247 (8)	−0.0027 (6)	−0.0014 (6)	0.0017 (6)
C4	0.0187 (7)	0.0245 (8)	0.0288 (9)	0.0034 (6)	0.0064 (6)	0.0064 (7)
C5	0.0246 (7)	0.0176 (7)	0.0244 (8)	0.0027 (6)	0.0054 (6)	0.0019 (6)
C6	0.0215 (7)	0.0169 (7)	0.0191 (8)	0.0009 (5)	0.0040 (6)	−0.0019 (6)
C7	0.0162 (6)	0.0125 (6)	0.0130 (7)	−0.0018 (5)	0.0037 (5)	−0.0012 (5)
C8	0.0224 (7)	0.0164 (7)	0.0142 (7)	0.0010 (5)	0.0060 (6)	0.0011 (5)
C9	0.0278 (7)	0.0193 (7)	0.0135 (7)	0.0011 (6)	0.0075 (6)	0.0000 (6)
C10	0.0236 (7)	0.0199 (7)	0.0200 (8)	0.0008 (6)	0.0092 (6)	−0.0022 (6)
C11	0.0261 (7)	0.0176 (7)	0.0195 (8)	0.0038 (6)	0.0082 (6)	0.0008 (6)
C12	0.0211 (7)	0.0179 (7)	0.0131 (7)	0.0024 (5)	0.0041 (5)	0.0019 (5)
C13	0.0150 (6)	0.0127 (6)	0.0134 (7)	−0.0014 (5)	0.0037 (5)	−0.0030 (5)
C14	0.0177 (6)	0.0173 (7)	0.0118 (7)	−0.0018 (5)	0.0011 (5)	0.0003 (5)
C15	0.0199 (7)	0.0169 (7)	0.0149 (7)	−0.0044 (5)	0.0047 (5)	0.0020 (5)
C16	0.0155 (6)	0.0151 (6)	0.0168 (7)	−0.0019 (5)	0.0027 (5)	−0.0030 (5)
C17	0.0173 (6)	0.0170 (7)	0.0145 (7)	0.0005 (5)	0.0009 (5)	−0.0002 (5)
C18	0.0191 (6)	0.0125 (6)	0.0136 (7)	0.0005 (5)	0.0044 (5)	0.0003 (5)
C19	0.0165 (6)	0.0234 (8)	0.0165 (7)	−0.0047 (6)	0.0010 (5)	0.0004 (6)
C20	0.0186 (7)	0.0364 (9)	0.0290 (9)	0.0006 (6)	0.0064 (6)	−0.0011 (7)
C21	0.0236 (7)	0.0239 (8)	0.0243 (8)	−0.0089 (6)	0.0045 (6)	−0.0019 (6)

### Geometric parameters (Å, º)

**Table:**

P1—C13	1.8176 (13)	C9—H9B	0.99
P1—C1	1.8321 (14)	C10—C11	1.524 (2)
P1—C7	1.8442 (14)	C10—H10A	0.99
P1—Se1	2.1288 (4)	C10—H10B	0.99
C1—C2	1.5329 (18)	C11—C12	1.532 (2)
C1—C6	1.5421 (19)	C11—H11A	0.99
C1—H1	1	C11—H11B	0.99
C2—C3	1.531 (2)	C12—H12A	0.99
C2—H2A	0.99	C12—H12B	0.99
C2—H2B	0.99	C13—C18	1.3908 (19)
C3—C4	1.524 (2)	C13—C14	1.3994 (19)
C3—H3A	0.99	C14—C15	1.3886 (19)
C3—H3B	0.99	C14—H14	0.95
C4—C5	1.524 (2)	C15—C16	1.4018 (19)
C4—H4A	0.99	C15—H15	0.95
C4—H4B	0.99	C16—C17	1.390 (2)
C5—C6	1.531 (2)	C16—C19	1.5143 (19)
C5—H5A	0.99	C17—C18	1.3928 (19)
C5—H5B	0.99	C17—H17	0.95
C6—H6A	0.99	C18—H18	0.95
C6—H6B	0.99	C19—C21	1.527 (2)
C7—C8	1.536 (2)	C19—C20	1.533 (2)
C7—C12	1.5392 (19)	C19—H19	1
C7—H7	1	C20—H20A	0.98
C8—C9	1.533 (2)	C20—H20B	0.98
C8—H8A	0.99	C20—H20C	0.98
C8—H8B	0.99	C21—H21A	0.98
C9—C10	1.524 (2)	C21—H21B	0.98
C9—H9A	0.99	C21—H21C	0.98
			
C13—P1—C1	105.70 (6)	C10—C9—H9B	109.5
C13—P1—C7	104.63 (6)	C8—C9—H9B	109.5
C1—P1—C7	107.81 (6)	H9A—C9—H9B	108
C13—P1—Se1	112.99 (5)	C9—C10—C11	110.45 (12)
C1—P1—Se1	113.56 (5)	C9—C10—H10A	109.6
C7—P1—Se1	111.56 (5)	C11—C10—H10A	109.6
C2—C1—C6	111.41 (11)	C9—C10—H10B	109.6
C2—C1—P1	111.05 (10)	C11—C10—H10B	109.6
C6—C1—P1	110.26 (10)	H10A—C10—H10B	108.1
C2—C1—H1	108	C10—C11—C12	111.36 (12)
C6—C1—H1	108	C10—C11—H11A	109.4
P1—C1—H1	108	C12—C11—H11A	109.4
C3—C2—C1	111.18 (12)	C10—C11—H11B	109.4
C3—C2—H2A	109.4	C12—C11—H11B	109.4
C1—C2—H2A	109.4	H11A—C11—H11B	108
C3—C2—H2B	109.4	C11—C12—C7	111.80 (12)
C1—C2—H2B	109.4	C11—C12—H12A	109.3
H2A—C2—H2B	108	C7—C12—H12A	109.3
C4—C3—C2	111.46 (13)	C11—C12—H12B	109.3
C4—C3—H3A	109.3	C7—C12—H12B	109.3
C2—C3—H3A	109.3	H12A—C12—H12B	107.9
C4—C3—H3B	109.3	C18—C13—C14	118.74 (12)
C2—C3—H3B	109.3	C18—C13—P1	119.72 (11)
H3A—C3—H3B	108	C14—C13—P1	121.31 (10)
C5—C4—C3	110.84 (12)	C15—C14—C13	120.29 (13)
C5—C4—H4A	109.5	C15—C14—H14	119.9
C3—C4—H4A	109.5	C13—C14—H14	119.9
C5—C4—H4B	109.5	C14—C15—C16	121.17 (13)
C3—C4—H4B	109.5	C14—C15—H15	119.4
H4A—C4—H4B	108.1	C16—C15—H15	119.4
C4—C5—C6	110.98 (12)	C17—C16—C15	117.95 (13)
C4—C5—H5A	109.4	C17—C16—C19	121.34 (13)
C6—C5—H5A	109.4	C15—C16—C19	120.70 (13)
C4—C5—H5B	109.4	C16—C17—C18	121.21 (13)
C6—C5—H5B	109.4	C16—C17—H17	119.4
H5A—C5—H5B	108	C18—C17—H17	119.4
C5—C6—C1	111.31 (12)	C13—C18—C17	120.56 (13)
C5—C6—H6A	109.4	C13—C18—H18	119.7
C1—C6—H6A	109.4	C17—C18—H18	119.7
C5—C6—H6B	109.4	C16—C19—C21	112.13 (12)
C1—C6—H6B	109.4	C16—C19—C20	110.84 (12)
H6A—C6—H6B	108	C21—C19—C20	110.70 (13)
C8—C7—C12	110.53 (11)	C16—C19—H19	107.7
C8—C7—P1	113.34 (9)	C21—C19—H19	107.7
C12—C7—P1	110.03 (9)	C20—C19—H19	107.7
C8—C7—H7	107.6	C19—C20—H20A	109.5
C12—C7—H7	107.6	C19—C20—H20B	109.5
P1—C7—H7	107.6	H20A—C20—H20B	109.5
C9—C8—C7	111.02 (11)	C19—C20—H20C	109.5
C9—C8—H8A	109.4	H20A—C20—H20C	109.5
C7—C8—H8A	109.4	H20B—C20—H20C	109.5
C9—C8—H8B	109.4	C19—C21—H21A	109.5
C7—C8—H8B	109.4	C19—C21—H21B	109.5
H8A—C8—H8B	108	H21A—C21—H21B	109.5
C10—C9—C8	110.89 (12)	C19—C21—H21C	109.5
C10—C9—H9A	109.5	H21A—C21—H21C	109.5
C8—C9—H9A	109.5	H21B—C21—H21C	109.5
			
C13—P1—C1—C2	169.50 (10)	C9—C10—C11—C12	−56.47 (16)
C7—P1—C1—C2	58.04 (11)	C10—C11—C12—C7	54.94 (16)
Se1—P1—C1—C2	−66.09 (11)	C8—C7—C12—C11	−53.92 (15)
C13—P1—C1—C6	−66.51 (11)	P1—C7—C12—C11	−179.86 (10)
C7—P1—C1—C6	−177.97 (9)	C1—P1—C13—C18	147.20 (11)
Se1—P1—C1—C6	57.90 (10)	C7—P1—C13—C18	−99.12 (12)
C6—C1—C2—C3	53.72 (17)	Se1—P1—C13—C18	22.43 (13)
P1—C1—C2—C3	177.05 (10)	C1—P1—C13—C14	−38.34 (13)
C1—C2—C3—C4	−55.38 (17)	C7—P1—C13—C14	75.35 (13)
C2—C3—C4—C5	57.02 (17)	Se1—P1—C13—C14	−163.10 (10)
C3—C4—C5—C6	−57.05 (17)	C18—C13—C14—C15	2.3 (2)
C4—C5—C6—C1	55.67 (17)	P1—C13—C14—C15	−172.26 (11)
C2—C1—C6—C5	−54.09 (16)	C13—C14—C15—C16	0.0 (2)
P1—C1—C6—C5	−177.88 (10)	C14—C15—C16—C17	−2.0 (2)
C13—P1—C7—C8	−61.65 (11)	C14—C15—C16—C19	177.00 (13)
C1—P1—C7—C8	50.53 (11)	C15—C16—C17—C18	1.8 (2)
Se1—P1—C7—C8	175.86 (8)	C19—C16—C17—C18	−177.24 (13)
C13—P1—C7—C12	62.67 (11)	C14—C13—C18—C17	−2.5 (2)
C1—P1—C7—C12	174.85 (9)	P1—C13—C18—C17	172.11 (11)
Se1—P1—C7—C12	−59.81 (10)	C16—C17—C18—C13	0.5 (2)
C12—C7—C8—C9	55.13 (15)	C17—C16—C19—C21	−124.22 (15)
P1—C7—C8—C9	179.19 (10)	C15—C16—C19—C21	56.82 (19)
C7—C8—C9—C10	−57.63 (16)	C17—C16—C19—C20	111.50 (16)
C8—C9—C10—C11	57.88 (16)	C15—C16—C19—C20	−67.46 (18)

### Hydrogen-bond geometry (Å, º)

Cg1 is the centroid of the C13–C18 benzene ring.

**Table:**

*D*—H···*A*	*D*—H	H···*A*	*D*···*A*	*D*—H···*A*
C1—H1···Se1^i^	1.00	3.09	4.0500 (14)	162
C9—H9*B*···*Cg*1^ii^	0.99	2.81	3.6471	143

Symmetry codes: (i) *x*, −*y*+1/2, *z*−1/2; (ii) *x*, −*y*−1/2, *z*−3/2.
